# Three-Dimensional *in Vitro* Cell Culture Models in Drug Discovery and Drug Repositioning

**DOI:** 10.3389/fphar.2018.00006

**Published:** 2018-01-23

**Authors:** Sigrid A. Langhans

**Affiliations:** Nemours Center for Childhood Cancer Research and Nemours Center for Neuroscience Research, Alfred I. duPont Hospital for Children, Wilmington, DE, United States

**Keywords:** three-dimensional cell culture, hydrogel, spheroid, high-throughput screening, extracellular matrix

## Abstract

Drug development is a lengthy and costly process that proceeds through several stages from target identification to lead discovery and optimization, preclinical validation and clinical trials culminating in approval for clinical use. An important step in this process is high-throughput screening (HTS) of small compound libraries for lead identification. Currently, the majority of cell-based HTS is being carried out on cultured cells propagated in two-dimensions (2D) on plastic surfaces optimized for tissue culture. At the same time, compelling evidence suggests that cells cultured in these non-physiological conditions are not representative of cells residing in the complex microenvironment of a tissue. This discrepancy is thought to be a significant contributor to the high failure rate in drug discovery, where only a low percentage of drugs investigated ever make it through the gamut of testing and approval to the market. Thus, three-dimensional (3D) cell culture technologies that more closely resemble *in vivo* cell environments are now being pursued with intensity as they are expected to accommodate better precision in drug discovery. Here we will review common approaches to 3D culture, discuss the significance of 3D cultures in drug resistance and drug repositioning and address some of the challenges of applying 3D cell cultures to high-throughput drug discovery.

## Introduction

With low success rates in clinical trials, drug discovery remains a slow and costly business. Currently, more than half of all drugs fail in Phase II and Phase III clinical trials due to a lack of efficacy and about another third of drugs fail due to safety issues including an insufficient therapeutic index (Arrowsmith and Miller, 2013). As attrition rates in drug discovery remain high, there is an urgent need for new technologies that accommodate better precision in drug discovery. Two of the most promising areas expected to improve the success rates in drug development are the advance of precision medicine with the prospect of new biomarkers and more precise drug targets and the availability of new preclinical models that better recapitulate *in vivo* biology and microenvironmental factors. Pioneered in the 1980's by Mina Bissell and her team performing studies on the importance of the extracellular matrix (ECM) in cell behavior, it is now well-accepted that culturing cells in three-dimensional (3D) systems that mimic key factors of tissue is much more representative of the *in vivo* environment than simple two-dimensional (2D) monolayers (Pampaloni et al., 2007; Ravi et al., 2015). While traditional monolayer cultures still are predominant in cellular assays used for high-throughput screening (HTS), 3D cell cultures techniques for applications in drug discovery are making rapid progress (Edmondson et al., 2014; Montanez-Sauri et al., 2015; Sittampalam et al., 2015; Ryan et al., 2016). In this review, we will provide an overview on the most common 3D cell culture techniques, address the opportunities they provide for both drug repurposing and the discovery of new drugs, and discuss the challenges in moving those techniques into mainstream drug discovery.

## The extracellular matrix (ECM) and other microenvironmental factors influencing the cell phenotype and drug response

### Extracellular matrix composition

**Figure 1:**
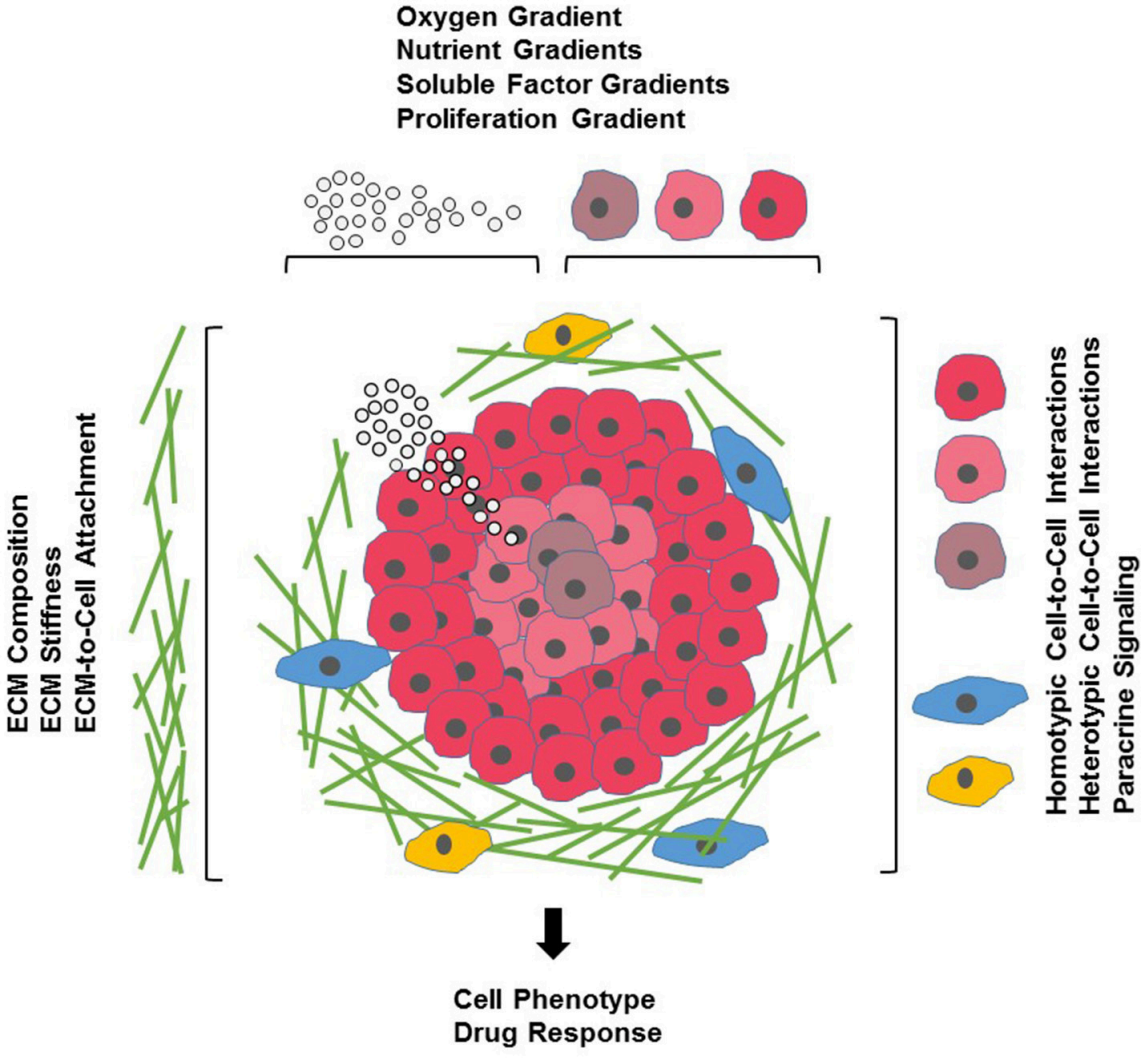
Cells and their microenvironment. Tissue-specific cells (red) encounter a complex microenvironment consisting of extracellular matrix (ECM) proteins and glycoproteins (green), support cells that mediate cell-cell interactions (blue), immune cells (yellow), and soluble factors (white spheres). The tissue microenvironment is further defined by physical factors such as ECM stiffness (indicated by increasing density of ECM proteins), and oxygen (indicated by red shading of tissue-specific cells) and nutrient and growth factor gradients (indicated by density of white spheres).

Cell-based assays are a crucial element of the drug discovery process. Compared to cost-intensive animal models, assays using cultured cells are simple, fast and cost-effective as well as versatile and easily reproducible. To date, the majority of cell cultures used in drug discovery are 2D monolayers of cells grown on planar, rigid plastic surfaces optimized for cell attachment and growth. Over the past decades, such 2D cultures have provided a wealth of information on fundamental biological and disease processes. Nevertheless, it has become clear that 2D cultures do not necessarily reflect the complex microenvironment cells encounter in a tissue (Figure 1). One of the biggest influences shaping our understanding of the limited physiological relevance of 2D cultures is the growing awareness of the interconnections between cells and the extracellular matrix (ECM) surrounding them. Earlier thought to mostly provide structural support, ECM components (for a comprehensive review of ECM constituents see Hynes and Naba, 2012) are now known to actively affect most aspects of cellular behavior in a tissue-specific manner. ECM molecules include matrix proteins (e.g., collagens, elastin), glycoproteins (e.g., fibronectin), glycosaminoglycans [e.g., heparan sulfate, hyaluronan (HA)], proteoglycans (e.g., perlecan, syndecan), ECM-sequestered growth factors [e.g., transforming growth factor-β (TGF-β), vascular endothelial growth factor (VEGF), platelet-derived growth factor (PDGF), hepatocyte growth factor (HGF)] and other secreted proteins (e.g., proteolytic enzymes and protease inhibitors). Dynamic changes in these components regulate cell proliferation, differentiation, migration, survival, adhesion, as well as cytoskeletal organization and cell signaling in normal physiology and development and in many diseases such as fibrosis, cancer and genetic disorders (Bonnans et al., 2014; Mouw et al., 2014). Thus, it is not surprising that the composition of the ECM along with its physical properties can also influence a cell's response to drugs by either enhancing drug efficacy, altering a drug's mechanism of action (MOA) or by promoting drug resistance (Sebens and Schafer, 2012; Bonnans et al., 2014).

Much of our knowledge on how the ECM can affect drug response and contributes to drug resistance comes from studies on the interaction of cancer cells and the tumor stroma in hematological malignancies and solid tumors. The microenvironment of a tumor, comprised of non-tumor cells (such as fibroblasts, endothelial cells, adipocytes, and immune cells) and ECM, is highly variable and depends on tumor type and location. Changes in ECM composition may influence drug response through altered local drug availability, by affecting expression of drug targets, or by changing intrinsic cellular defense mechanisms such as increased repair upon DNA damage or evasion of apoptosis (Sebens and Schafer, 2012; Junttila and de Sauvage, 2013; McMillin et al., 2013; Holle et al., 2016). Interactions between cells and ECM can also lead to a heterogenous drug response with matrix-attached outer cells being drug resistant and matrix-deprived cells in the core of the tumor being sensitive (Muranen et al., 2012). It is well documented that the adhesion between cells and ECM proteins, mediated mainly by the integrin system of transmembrane receptors, is an important factor modulating the response to chemotherapeutics and targeted therapies in oncology or to other therapeutic approaches such as immunotherapy, radiation or radiochemotherapy (Holohan et al., 2013; Holle et al., 2016; Dickreuter and Cordes, 2017; Jiang et al., 2017). Other ECM components such as heparan sulfate (Lanzi et al., 2017), hyaluronic acid (a physiological ligand for the cell surface receptor CD44 often found in cancer stem cell niches) (Bourguignon, 2016), soluble factors such as matrix metalloproteinases (MMPs) (Candido et al., 2016), tissue inhibitors of metalloproteinases (TIMPs) (DeClerck, 2000) and various cytokines and growth factors (Holohan et al., 2013), all have been shown to alter drug response and mediate drug resistance in cancer. For this reason, modern drug strategies take advantage of targeting the interactions between tumor cells and tumor-promoting microenvironmental factors. Such an approach requires cancer models that more faithfully mimic a tumor's microenvironment and makes cancer drug discovery the fastest growing application for 3D cell cultures. However, changes in drug response in response to ECM remodeling are not limited to tumors. For example, insulin resistance in obesity is known to be affected by EMC remodeling in adipose tissue (Williams et al., 2015; Lin et al., 2016) and cytokines, growth factors and ECM proteins play significant roles in the development of fibrotic diseases in many tissues including liver, lung and kidney (Wynn and Ramalingam, 2012; Bonnans et al., 2014; Handorf et al., 2015), requiring genuine culture models that can mimic *in vivo* conditions for such diseases.

### Matrix stiffness

The ECM is characterized by its biochemical composition and its physical and mechanical properties with tissue stiffness being important for the maintenance of homeostasis (Handorf et al., 2015). Changes in ECM composition are often accompanied by changes in physical cues such as rigidity, leading to bidirectional changes in cells and the ECM (Hynes, 2014; Alcaraz et al., 2017; Zollinger and Smith, 2017). Cells can respond to mechanical forces through changes in cell division, morphogenesis, migration, signaling, gene expression, ion channel gating, or vesicle formation and by further remodeling of the ECM (Hamill and Martinac, 2001; Eyckmans et al., 2011; Tyler, 2012). The relationship between tissue stiffness and cell phenotype and cell function has been well described in tumors and in the brain. Tumors are usually stiffer than surrounding healthy tissue and tissue stiffness can contribute to drug resistance (Holle et al., 2016; Bordeleau et al., 2017; Lin et al., 2017). In the brain, tissue stiffness is a major factor in development and brain plasticity (Tyler, 2012; Barnes et al., 2017). With brain being one of the softest tissues in the body, its ECM is characterized by a relative low abundance of matrix proteins and a high prevalence of glycosaminoglycans, proteoglycans and glycoproteins, some of which are brain-specific. This leads to cell-ECM interactions that are not only mediated by integrins, but also by a variety of tissue-specific non-integrin receptors predominantly found in neurons and glial cells (Barros et al., 2011). The stiffness of brain regions varies in normal brain and mechanical properties change with age (Happel and Frischknecht, 2016) and in a wide range of neurological disorders, including multiple sclerosis, Alzheimer's disease, epilepsy and schizophrenia (Tyler, 2012; Murphy et al., 2016). The unique composition of brain ECM together with its well-established roles in neurotransmitter function and receptor turnover, ion channel activity, synaptic plasticity and dendritic spine formation (Frischknecht and Gundelfinger, 2012) gives rise to the need of cell culture models that reflect the complexity of the brain ECM surrounding neurons and glial cells such as astrocytes, oligodendrocytes and microglia. With drug failure in neurological disorders exceeding that in many other diseases, 3D culture models are promising technologies to meet the challenge of developing more realistic *in vitro* disease models for CNS drug discovery.

### Concentration gradients

Within a tissue, concentration gradients exist for oxygen, pH and soluble components such as nutrients and effector molecules as well as cellular metabolites. These natural gradients are influenced by the proximity to blood vessels, by the diffusion of molecules through the ECM, and thus, the composition of the ECM, and by the extent of cellular metabolism that regulates oxygen and nutrient consumption and the production of cellular waste products. Molecular concentration gradients affect various cell behavior, including cell motility, cell migration, and cell signaling and are important in chemotaxis and morphogenesis in normal development and in wound healing. Depending on the proximity to a blood vessel, small, avascular tumors or metastasis often display a gradient in oxygen levels leading to a proliferative zone and a hypoxic core with quiescent cells that are more resistant to chemotherapy, immunotherapy and radiation therapy (Herrmann et al., 2008; Carrera et al., 2010; Chouaib et al., 2017). Traditional monolayer cultures are not amenable to studies of oxygen or nutrient gradients as all cells are homogenously exposed to the tissue culture medium. In *in vivo* models, hypoxia occurs naturally or can be induced. However, these are complex models and are associated with high cost and variability. Thus, 3D cultures such as spheroids, cells encapsulated into 3D matrices and microdevice platforms provide opportunities to understand oxygen, growth factor and nutrient-mediated mechanisms leading to changes in cell phenotype and alterations in drug response.

### Stromal cells

*In vivo*, cells are not only surrounded by ECM and ECM-associated signaling molecules, but connective tissue also contains stromal cells, including mesenchymal supporting cells such as fibroblasts or adipocytes in epithelial tissue, glial cells in neuronal tissue, cells of the surrounding vasculature and cells of the immune system. Interactions with stromal cells can regulate surrounding epithelial and neuronal tissue, contribute to disease progression and influence the therapeutic response of cells. In tumors, carcinoma-associated fibroblasts can stimulate tumor cell growth, induce angiogenesis and promote inflammation and stromal cells within cancer stem cell niches drive drug resistance (Egeblad et al., 2010; Jones et al., 2016; Prieto-Vila et al., 2017). During the angiogenic switch of tumors, vasculogenesis involves the recruitment of endothelial cells, perivascular cells and bone marrow-derived cells, a process that cannot only be exploited for cancer therapy but for which the stromal environment is an important factor in regulating drug response (Crawford and Ferrara, 2009; Egeblad et al., 2010; Gacche, 2015; Lopes-Bastos et al., 2016; De Palma et al., 2017). Inflammatory cells are components of normal tissue and play an important role during normal development. At the same time, stromal immune cells contribute to a variety of diseases ranging from diabetes to artherosclerosis, fibrosis, cancer and neurodegeneration (Wynn et al., 2013). While targeting stromal immune cells is a promising strategy for the development of novel therapeutics for a wide range of diseases (Kersh et al., 2018) and recently has gained wide attention in cancer drug discovery and for the therapy of neurological diseases (Villoslada et al., 2008; Pitt et al., 2016; Alsaab et al., 2017; Chitnis and Weiner, 2017; Kabba et al., 2017; Pogge von Strandmann et al., 2017), immune cells also modulate therapeutic response to drugs not directly targeting the immune system (Kersh et al., 2018). In drug discovery, *in vitro* modeling of such complex interactions will require multicellular 3D tissue models with organoids currently being at the forefront for disease modeling, drug screening and drug development.

### 3D cell culture models

**Figure 2:**
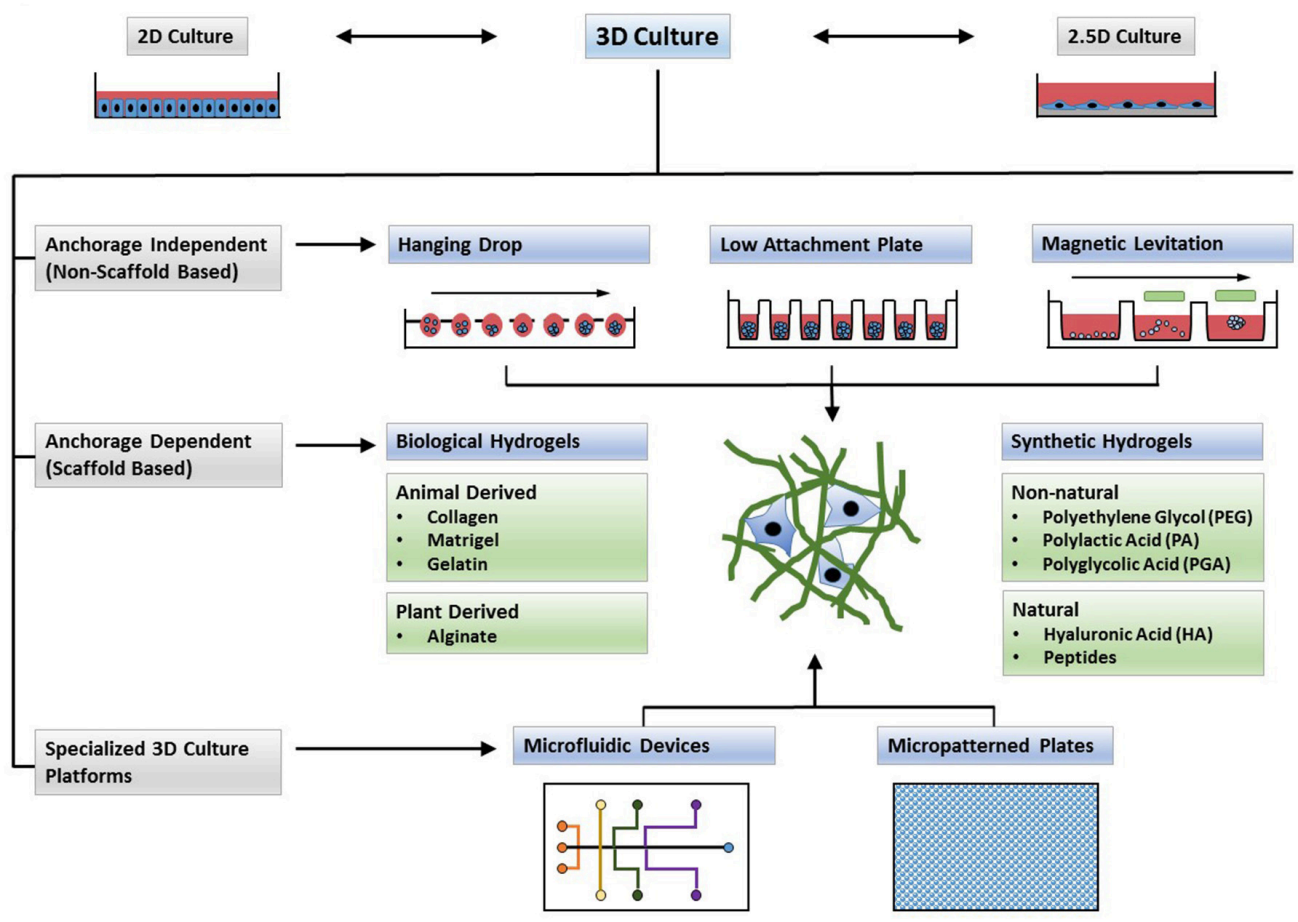
Types of 3D cultures. In contrast to 2D monolayers or 2.5D cultures in which cells are plated on top of a thick layer of extracellular matrix (ECM), 3D cultures form complex structures. Cells may form spheroids (anchorage independent systems) or can be encapsulated in cell culture scaffolds (anchorage dependent). Microfluidic devices and micropatterned plates with ECM components, and cultures in which formed spheroids are embedded in ECM scaffolds, can form hybrid culture systems that combine the advantages of both systems to form a complex microenvironment for 3D cell culture.

An ideal 3D culture model would simulate a tissue-specific physiological or pathophysiological disease-specific microenvironment where cells can proliferate, aggregate and differentiate. Such a model would include cell-to-cell and cell-to-ECM interactions, tissue-specific stiffness, oxygen, nutrient and metabolic waste gradients, and a combination of tissue-specific scaffolding cells (Griffith and Swartz, 2006). Most 3D culture techniques, often categorized into non-scaffold, anchorage-independent and scaffold-based 3D culture systems as well as hybrid 3D culture models in which formed spheroids are incorporated into a 3D polymeric scaffold (Ho et al., 2010), currently do not meet all of the above criteria but rather have their own strengths and limitations. Thus, one will need to choose the most appropriate 3D cell culture model for a specific application. For example, scaffold-based models more readily mimic cell-to-ECM interactions while non-scaffold based spheres of certain size are more amenable to cellular and physiological gradients. Traditional 3D cell culture models such as spinner flasks (Sutherland et al., 1970) or gyratory rotation devices (Goodwin et al., 1993; Breslin and O'Driscoll, 2013) provide large-scale methods to generate 3D spheres but lack the possibility for miniaturization and are not compatible with HTS methods. Many of the newer 3D culture systems (Figure 2) allow for microscale 3D cultures and provide a first step toward developing technologies for 3D cultures that are compatible with automated high-throughput screening allowing for the discovery of new drug candidates or repositioning of known drugs in physiologically more relevant cell cultures.

### Anchorage-independent technologies

Scaffold-free 3D culture methods rely on the self-aggregation of cells in specialized culture plates, such as hanging drop microplates, low adhesion plates with ultra-low attachment coating that promotes spheroid formation and micropatterned plates that allow for microfluidic cell culture. Spheroids, and in particular multicellular spheroids (Mueller-Klieser, 1987; Sutherland, 1988), recapitulate physiological characteristics of tissues and tumors with regard to cell-cell contact, and if synthesizing their own ECM, allow for natural cell-matrix interactions. Spheroid size depends on the initial number of cells seeded and spheroids can be grown to a size where they display oxygen and nutrient gradients similar to tissue (Cukierman et al., 2001; Doublier et al., 2012; Ekert et al., 2014). Neurospheres are spheres of mixed cultures of progenitors, neuronal and glial cells. While they consist mostly of poorly differentiated cells, neurospheres allow for interactions between different cell types of the CNS. These interactions play important roles in neuronal differentiation, in the conversion of toxic compounds into active metabolites, and in the secretion of apoptotic factors upon drug treatment; thus neurospheres allow for CNS drug discovery in a physiologically more relevant culture system (Campos, 2004; Moors et al., 2009). Tumor spheroid models derived from established cell lines or obtained through *ex vivo* propagation of tumors from individual patients (tumor organoids) have been established for a variety of tumor types. Tumor spheroids from patients retain their genome over time and can be used to perform drug screens and facilitate drug development in a patient-centered manner (Stadler et al., 2015; Nath and Devi, 2016; Pauli et al., 2017; Weeber et al., 2017). The disadvantage of spheroid cultures, whether tumor spheroids or neurospheres, however, is the need for much optimization of culture conditions to obtain uniform spheroids to enhance reproducibility and of size not too large to prevent insufficient nutrient supply and necrosis.

### Hanging drop microplates

Hanging drop cultures are a well-known 3D culture technology taking advantage of self-aggregation of cells into spheroids when a surface is not available for cell attachment. Hanging drops can be created in specialized plates with open, bottom-less wells that are designed for the formation of a small droplet of media. The droplet is big enough for cells suspended in the medium to aggregate but small enough to prevent it from being dislodged during manipulation. Cultured for several days, cells in hanging drop plates (HDP) can form spheres that may represent tumor layers in the vicinity of a capillary—a peripheral layer with proliferating cells due to the closeness to the supply of oxygen and nutrients, underlaid by an intermediate layer with quiescent cells and an inner necrotic core. With this, spheres can mimic inward diffusion to form oxygen and nutrient gradients, model in- and outward diffusion of regulatory molecules, and provide a reservoir for the accumulation of waste, accompanied by low pH. The spheroid size can be controlled by the initial number of cells suspended in the drops but may require transfer from an HDP plate to a second non-attachment propagation plate with higher media volume to ensure suitable culture conditions with adequate nutrient supply and pH over longer times and to allow for the formation of bigger spheres. Multi-cellular spheres may be created by co-suspending several cell types or by consecutive addition of different cell types to promote the formation of separate cell layers. Embedding of formed spheres into ECM-like scaffolds allows for the modeling of cell adhesion of the outer layers of the spheres with ECM components surrounding tumor tissues. Spheroids formed in HDP plates as well as in low adhesion plates described below have evolved into a common 3D cell culture technology in cancer research, examples of which are described in a comprehensive review by Stadler et al. (2015). Hanging drop cultures have also found applications in toxicity testing in hepatocytes (Shri et al., 2017) or in engineering cardiac spheroids (Chitnis and Weiner, 2017). In a recent study, human primary or induced pluripotent stem cell (iPSC)-derived cardiomyocytes were co-cultured with endothelial cells and fibroblasts in a ratio similar to that found *in vivo*. The cardiac spheroids mimicked important *in vivo* features of the human heart biochemically and pharmacologically offering a 3D cell culture model to study toxic effects in human heart tissue (Polonchuk et al., 2017).

### Spheroid microplates with ultra-low attachment coating (low adhesion plates)

Similar to HDP cultures, spheroid microplates with round, tapered or v-shaped bottoms, take advantage of the lack of cell attachment surfaces to promote aggregation of cells and spheroid formation. In contrast to HDP cultures, however, a transfer of spheroids to a different plate for prolonged culture or experimental procedures is often not necessary. 96- or 384-well plates have an initial higher volume capacity than droplets and eliminate steps that would involve the need for manipulating spheroids. Low adhesion plates are often made of polystyrene and treated with hydrophilic or hydrophobic coatings like the non-adherent polymer poly-HEMA (Ivascu and Kubbies, 2006) or natural polymers such as agarose (Friedrich et al., 2009; Li et al., 2011). The coating reduces the attachment of cells to the point that they preferably aggregate with each other to form spheroids. Because of their larger volume, low adhesion plates are well suited for multicellular culture and are frequently used for studies in tumor cells. For example, when grown on chitosan-hyaluronan substrates, multicellular spheres formed from two non-small cell lung cancer (NSCLC) cells exhibited more stem-cell like characteristics, an increase in cell motility and expression of markers for epithelial-to-mesenchymal transition (EMT), and the stem-cell like cells displayed multidrug resistance when compared to 2D cell cultures (Huang and Hsu, 2014). Spheroids, but not 2D cultures, of patient-derived breast cancer cells simulated tumor characteristics *in vivo* such as hypoxia, dormancy, anti-apoptotic features and drug resistance (Imamura et al., 2015). Another recent study took advantage of forming brain tumor spheres in a mixed-neuronal culture environment by co-culturing brain tumor cells with human fetal brain tissue to develop an *in vitro* model for drug delivery assessments in pediatric medulloblastoma (Iskandar et al., 2015).

### Magnetic levitation

Magnetic cell levitation is an emerging technique for the formation of spheroids. To generate spheroids, cells are preloaded with magnetic nanoparticles and then, using an externally applied magnetic field, are floated toward the air/liquid interface within a low adhesion plate to promote cell-cell aggregation and spheroid formation. Magnetic levitation has been used to generate spheroids from cells of various tissue, to form multicellular mesenchymal stem cells spheroids and for tissue engineering (Souza et al., 2010; Haisler et al., 2013; Tseng et al., 2013; Lewis et al., 2016, 2017). Magnetically levitated human glioblastoma cells closely recapitulated *in vivo* protein expression observed in human glioblastoma tumor xenografts (Souza et al., 2010) and recently, a high-throughput assay for toxicity screening in 3D cell cultures using magnetic levitation was described, making this a promising new technique in drug discovery (Timm et al., 2013).

### Scaffold-based technologies

Scaffold-based culture technologies provide physical support, ranging from simple mechanical structures to ECM-like matrices, on which cells can aggregate, proliferate and migrate. In 2.5D cultures, cells are grown on top of a thick layer of ECM proteins that allows for tissue-specific differentiation of a variety of cells. Nevertheless, such cultures do not necessarily represent an *in vivo* environment as cells are still exposed to tissue culture medium and lack ECM contact on the surface (Shamir and Ewald, 2014). In scaffold-based 3D cultures, cells are embedded into the matrix and the chemical and physical properties of the scaffold material will influence cell characteristics. Scaffolds can be of biological origin or they can be synthetic engineered to mimic key properties of ECM such as stiffness, charge or adhesive moieties. In some synthetic scaffolds, growth factors, hormones or other biologically active molecules can be encapsulated to enhance cell proliferation or to promote a specific cell phenotype. Thus, when selecting a 3D cell culture scaffold for a certain application, one will need to consider properties of the material that define physical factors such as porosity, stiffness and stability in culture as well as biological properties such as cell compatibility or adhesiveness (Caliari and Burdick, 2016). Hard polymers can provide the physical support found in a specialized tissue, such as skin, tendons or bone and micropatterned surface microplates can be designed for specific applications such as support of cell networking.

### Hydrogel scaffolds of biological origin

Hydrogels are networks formed from dilute polymer chains with given structure and properties, obtained either by intermolecular (polymer network) or by interfibrillar crosslinks (supramolecular fibrillary hydrogel network) (Tibbitt and Anseth, 2009; Li and Deming, 2010; Yan and Pochan, 2010; Worthington et al., 2015). Although hydrogels display solid-like material properties in the quiescent state, with over 95% of water by volume, they can indeed provide a cell-liquid interface (Sathaye et al., 2015). Hydrogels may come from natural sources or can be synthetic, with the possibility of mixing different hydrogel materials to obtain hybrid hydrogels possessing new physical and biological properties. Hydrogels from natural sources such as collagen, fibrin or Matrigel are biocompatible, have natural adhesive properties and sustain many physiological cell functions—resulting in high cell viability, controlled proliferation or controlled differentiation, and often a cell phenotype typically observed *in vivo*. Collagen, with type I collagen being the most abundant form, is the most widely used ECM protein for 3D cell culture (Glowacki and Mizuno, 2008; Pathak and Kumar, 2011; Orgel et al., 2014). Altering collagen concentrations or gelation temperature leads to changes in collagen hydrogel stiffness allowing for controlled changes in cell proliferation (Kutschka et al., 2006; Doyle et al., 2015) and, depending on collagen stiffness, pancreatic cancer cells respond differently to gemcitabine (Puls et al., 2017). Collagen, as well as Matrigel, facilitate cell attachment through integrin receptors which leads to activation of cell signaling pathways that control cell survival, growth and differentiation (Yang et al., 2004; Kutschka et al., 2006) and can modulate the response to therapeutic approaches, including chemotherapy, immunotherapy and radiation (Holle et al., 2016; Dickreuter and Cordes, 2017). For example, comparison of drug response profiles of breast, prostate and lung cancer cell lines revealed clear differences in dose response curves to docetaxel and fulvestrant when cells were grown in collagen and compared to 2D cultures or other 3D culture systems such as low attachment plates and other natural scaffolds, including alginate and Matrigel (Stock et al., 2016). Multiple myeloma cells cultured in a Matrigel-based human bone marrow-like microenvironment provide a system for preclinical testing of chemotherapeutics that take into account adhesion-mediated mechanisms of drug resistance (Kirshner et al., 2008) and Matrigel-embedded 3D-tumoroids derived from tissue of patients with colorectal cancer and lung cancer can provide a 3D culture system for drug testing that contains not only tumor cells but also immune cells from surrounding tissues (Finnberg et al., 2017). Matrigel and similar products are a gelatinous mixture of proteins and growth factors secreted by Engelbreth-Holm-Swarm mouse sarcoma cells (Kleinman and Martin, 2005). Since Matrigel is minimally processed, it provides a good mimic of *in vivo* ECM (Poincloux et al., 2011; Wong et al., 2011). Matrigel consists mostly of laminin and collagen as well as a small fraction of entactin (a basement membrane glycoprotein) and contains several growth factors, including EGF (epidermal growth factor), bFGF (basic fibroblast growth factor), NGF (nerve growth factor), PDGF, IGF-1(insulin-like growth factor 1) and TGF-β (Hughes et al., 2010). Since Matrigel is processed from natural sources, batch-to-batch variability of the purified scaffold may interfere with pharmacological studies of drug response. A growth-factor reduced formulation of Matrigel is available, allowing for a 3D culture setup with more defined properties (Wallace and Rosenblatt, 2003). Nevertheless, due to the natural origin and manufacture from live tissue, collagen and Matrigel are complex scaffolds that contain besides their major constituents many other components and are therefore chemically not well defined (Hughes et al., 2010; Gill and West, 2014). While collagen and Matrigel both support enhanced interaction of cells with ECM proteins, due to their different composition, cells embedded into collagen or Matrigel can display different phenotypes (Borlak et al., 2015). Collagen and Matrigel are available in liquid form and require handling at cold temperatures to avoid premature gelation. The need for handling these hydrogels at low temperatures makes them unsuitable for common liquid handling equipment used for high-throughput screens in drug discovery (Ryan et al., 2016; Worthington et al., 2017). Fibrin is obtained through polymerization of fibrinogen, a plasma protein, and is a natural polymer formed during wound coagulation. While it has been used for *in vitro* cultures, including angiogenesis studies, biomechanical studies and mesenchymal stem cell culture, its high susceptibility to protease-mediated degradation limits its use in long-term cultures (Ahmed et al., 2008; Anitua et al., 2013; Kural and Billiar, 2013; Brown and Barker, 2014; Caliari and Burdick, 2016). Gelatin is a partial thermally and chemically degraded product of collagen and can be stabilized by covalent modification. The possibility of covalent linking of functional groups also allows for the production of specialized gelatin gels, that for example can be photoreactive (Banks et al., 2015) or oxygen-controllable to form hypoxic gradients in 3D cultures (Lewis et al., 2017). Another natural hydrogel is alginate that is isolated from the cell walls of brown algae. The mechanical properties and rapid degradation of the alginate hydrogel somewhat limits its application for 3D cultures but alginate hydrogels have found their use as 3D-printed scaffolds for specialized tissue such as vascular tissue, bone and cartilage (Axpe and Oyen, 2016; Joddar et al., 2016; Silva et al., 2017).

### Synthetic hydrogels

When well-designed, synthetic hydrogels are ideal materials to use as 3D cell culture scaffolds as they can mimic biological properties of ECM, be functionalized with defined adhesive moieties, include proteolytic sites and encapsulate growth factors. At the same time, they are chemically and physically well-defined and often have tunable mechanical properties to achieve a desired stiffness or porosity (Worthington et al., 2015; Zhang and Khademhosseini, 2017). Synthetic hydrogels can be categorized into non-natural and natural polymers. Polyethylene glycol (PEG), polylactic acid (PA), polyglycolic acid (PGA) and other unnatural polymer hydrogels (Raeber et al., 2005; Zhang and Khademhosseini, 2017) have the advantage of being comparatively inexpensive, are relatively inert, have reproducible material properties that are usually easy to tune through synthesis or crosslinking, and are reproducible, thereby supporting the acquisition of consistent results. On the other side, however, unnatural polymers lack adhesive moieties found in natural ECM and require crosslinking of biological peptides to the scaffold to improve functionality (Weber et al., 2007; Kraehenbuehl et al., 2008). PEG gels and their derivatives have been used in a variety of 3D cell culture applications including stem cell differentiation, cell invasion and angiogenesis (Lutolf et al., 2003; Moon et al., 2010; Zhu, 2010; Caiazzo et al., 2016). Synthetic natural polymers share with non-natural polymers the advantage of consistent and tunable material properties for reproducible results. Due to the biological nature of their naturally occurring moieties, natural synthesized hydrogels are highly compatible with encapsulation of cells for 3D cell culture. However, material cost can be high due to complex chemical synthesis requirements. One of the best characterized natural hydrogels is hyaluronic acid (HA)—a glycosaminoglycan that can be modified with functional groups, allowing for the formation of hydrogels with diverse properties for a wide range of applications (Burdick and Prestwich, 2011; Baeva et al., 2014; Goubko et al., 2014). Small peptide-based hydrogel materials are an evolving field in materials science and provide a new 3D cell culture technology that is amenable to drug discovery studies. Recently, a peptide-based 3D mesenchymal stem cell co-culture model of the multiple myeloma bone marrow niche has been described where patient-derived tumor cells displayed resistance to chemotherapeutics that was reflective of clinical resistance and thus, may provide a technology platform for drug testing and precision medicine in multiple myeloma patients (Jakubikova et al., 2016). While diverse in primary structure, the peptides have a similar overall ability to form nanofibrillar structures with intramolecular folding and intermolecular assembly triggered by physical or chemical cues. Peptide hydrogels are highly versatile and material properties can be modulated by substituting amino acids, extending or shortening the peptide sequence, or by the addition of functional epitopes (Branco et al., 2011; Yang and Zhao, 2011; Li et al., 2013; Wang et al., 2013; Worthington et al., 2015). Current peptide hydrogels that are most successfully used for 3D culture (reviewed in Worthington et al., 2015) are: the yeast-derived peptides EAK16 and RADA16 (Zhang et al., 1992, 2005); the peptides Fmoc-FF (Fluorenylmethoxycarbonyl-diphenylalanine) and Fmoc-RGD (Fluorenylmethoxycarbonyl arginine–glycine–aspartic acid) (Jayawarna et al., 2006; Mahler et al., 2006; Smith et al., 2008; Orbach et al., 2009; Zhou et al., 2009); the peptide hydrogel h9e that is based on the fusion of functional domains from a silk protein and a human calcium channel (Huang and Sun, 2010; Huang et al., 2011, 2013); FEFK and FEFKEFK, which form hydrogels in the presence of a metalloprotease (Toledano et al., 2006; Guilbaud et al., 2010); and the MAX1 peptide that gelates under physiological conditions, and like h9e, has shear-thinning properties (Schneider et al., 2002; Haines-Butterick et al., 2007). A single acid substitution derivative of the MAX1 peptide, MAX8 (Haines-Butterick et al., 2007), with reduced gelation times has recently been reported to be compatible with liquid handling equipment making it suitable for high-throughput drug discovery (Worthington et al., 2017).

### Polymeric hard scaffolds, micropatterned surface microplates, and microfluidic devices

Microfabrication technology allows for the fabrication of an endless array of imprinted micropatterns on the surface of plates. Coated for low adhesion, the micro-patterned plates can be designed to promote cell-to-cell adhesion for scaffold-free microsphere formation within the confinement of a microspace. Micropatterned plates can also be manufactured to provide a scaffold-based 3D culture environment to promote cell attachment for the formation of contiguous networks along surfaces and organoids. Polymeric pre-fabricated scaffolds, such as porous discs, electrospun scaffolds or orthogonally layered polymers, are physical supports that can be an inert matrix or designed to mimic *in vivo* ECM on which cells can attach, migrate or fill scaffold compartments to form 3D cultures carrying a geometric configuration (Knight et al., 2011). Currently, the most common applications for such scaffolds are for tissue regeneration recreating the natural physical and structural environment of bone, ligaments and cartilage, for skin, vascular, skeletal muscle or CNS tissue and for preclinical *in vitro* 3D culture testing of tumoroids or engineered tissues (organoids). In particular, tumoroids derived from patient samples are promising techniques for drug screening and drug development in precision medicine (Stadler et al., 2015; Nath and Devi, 2016; Pauli et al., 2017; Weeber et al., 2017). Recently, a microspun 3D fibrous scaffold for tumoroid formation was developed as a platform for andicancer drug development (Girard et al., 2013). When compared to 2D monolayers, HepG2 liver cells grown on 3D porous polystyrene scaffolds had greater cell viability and formed bile canaliculi, and at the same time were less susceptible to cytotoxic compounds (Bokhari et al., 2007). Microfluidic devices are designed for cell cultures under perfusion and allow for steady supplies of oxygen and nutrients while at the same time removing waste. Microfluidic devices can be built to mimic shear forces found *in vivo* in cells that are exposed to blood flow like endothelial cells. A barrier between compartments can be physically incorporated into the device or it can consist of a non-physical barrier such as a supporting matrix mimicking ECM. Microfluidic devices allow for the continued application of drugs or other soluble molecules such as growth factors, or the exchange of fluid between different compartments that may harbor different types of cells. Microfluidic devices can be used for long-term tumoroid cultures (Aw Yong et al., 2017) and Montanez-Sauri, et al recently described an automated microfluidic ECM screening platform with the capability for small molecule screening (Montanez-Sauri et al., 2013). Microengineering of microfluidic devices also allows for the development of organ-on-a-chip platforms with 3D tissue models having been described for a variety of organs including skin, muscle, liver and neural tissue, bridging *in vitro* cell culture and *in vivo* animal models. With the advancement of these 3D culture technologies, organs-on-a-chip are poised to provide advanced tools for drug development and high-throughput screening in the future (Alépée et al., 2014; Pamies et al., 2014; Abaci et al., 2017).

### Organoids

Originally, the term organoid referred to primary cultures of tissue fragments separated from the stroma within 3D gels to from organ-like structures (Simian and Bissell, 2017). Over the past decade, the term organoid has broadened and now encompasses a variety of tissue culture techniques that result in self-organizing, self-renewing 3D cultures derived from primary tissue, embryonic stem cells, or induced pluripotent stem cells that have a similar functionality as the tissue from which the cells originate (Lancaster and Knoblich, 2014; Shamir and Ewald, 2014; Clevers, 2016; Fatehullah et al., 2016; Kretzschmar and Clevers, 2016; Simian and Bissell, 2017). While current organoid cultures often still face limitations, such as the lack of a native microenvironment (e.g., ECM composition, growth factor gradients) or the lack of interactions with immune cells and, consequently, the inability to model immune responses, organoids derived from human cells have the potential to provide near-physiological models to study human development and human diseases. With this, more advanced organoid cultures will allow for developing screening platforms for drug discovery that are more cost-effective than animal models and can provide precise models of human diseases that cannot be recapitulated in animals. Organoid cultures have been described for a variety of organs, including various normal tissue and disease models of the digestive tract, prostate, lung, kidney and the brain (Clevers, 2016; Fatehullah et al., 2016; Dutta et al., 2017). Currently, transcriptome profiling is one of the most common downstream applications of organoids but applications in drug discovery and precision therapy are evolving (Fatehullah et al., 2016; Liu et al., 2016). For example, kidney organoids have been used for toxicity screening in response to cisplatin (Takasato et al., 2015), an organoid model of cystic fibrosis for drug screening has been described (Dekkers et al., 2013) and a high-throughput platform for intestinal stem cell niche co-cultures has been developed (Gracz et al., 2015). Beyond such organoids, tumoroids derived from patient cancer tissues that contain tumor cells and stroma cells of the tumor microenvironment are poised to provide advanced and more realistic 3D culture platforms for personalized drug evaluation and development (Xu et al., 2014; Stadler et al., 2015; Pauli et al., 2017). Further, tumoroids that retain tissue identity, paired with organoids of adjacent healthy tissue, can lay the foundation to construct tumor organoid biobanks as repository for drug screening and development (van de Wetering et al., 2015).

### Applications of 3D cultures in drug discovery and drug repositioning

In the past, cell-based drug discovery emphasized chemical screens in well-characterized cell monolayers, and mostly in cancer drug discovery, in large panels of authenticated cell lines (Smith et al., 2010; Barretina et al., 2012). However, in recent years, 3D cell culture systems that model *in vivo* microenvironmental aspects, and are therefore expected to yield results with higher predictive value for clinical outcome, are becoming more prominent in drug discovery. In addition, authentic 3D cell culture models using human cells can circumvent drawbacks of mouse models that, aside from the high cost and ethical considerations, are not always able to accurately recapitulate human diseases or capture side effects of drugs such as liver toxicity (Sivaraman et al., 2005; Aparicio et al., 2015). In order to enhance the drug discovery process, aid in the development of new pharmacological approaches or to be useful *in vitro* toxicity screens, 3D cell culture models will need take into account that the response to a broad spectrum of drugs varies not only with a particular cell line or tumor type, but also with its surrounding stroma. The response to therapeutic compounds may range from drug resistance to enhanced sensitivity based on tissue-specific composition of the ECM, the interaction with stromal cells and the presence of immunomodulatory molecules (Turley et al., 2015; Johansson et al., 2016; Stock et al., 2016). While research into new 3D culture technologies that take into account the functional unit of tissues such as organoids has gained great momentum (Lancaster and Knoblich, 2014; Shamir and Ewald, 2014; Clevers, 2016; Kretzschmar and Clevers, 2016; Simian and Bissell, 2017), much work remains to be done to develop systems that accurately represent *in vivo* conditions and disease pathology. At the same time, 3D cell cultures open up the door to model the cell culture environment to promote a desired cell behavior. Models focused on enhanced cell motility, induction of cell dormancy, promotion of cell differentiation in epithelial cells and neurons, the support of stem cell-like properties or a desired microenvironment like that of a metastatic niche (Valastyan and Weinberg, 2011; Sleeman, 2012), enable the possibility of more specifically targeting certain cell behavior in drug discovery. In addition, cancer drug discovery combining 3D cell culture technology with primary patient-derived tumor cells (Ma et al., 2015), and molecular profiling data or the formation of 3D organoid banks of tumor cells that are representative of molecular tumor subtypes (van de Wetering et al., 2015), may open the door for preclinical screening of a personalized panel of drug candidates to improve outcome and reduce side effects of cancer therapy.

### Limitations of 3D cell culture technologies in drug discovery

High-throughput screening (HTS) to determine the biological or biochemical activity of chemically diverse small compound libraries or high-content screening (HCS) used to identify compounds that alter a cell's phenotype is an integral part of drug discovery. Application of 3D cell culture in HTS and HCS, however, remains a challenge (Rimann and Graf-Hausner, 2012; Edmondson et al., 2014; Montanez-Sauri et al., 2015; Ryan et al., 2016). Aside from the question of biological and disease relevance, labor intensiveness and material cost, scalability to 384- and 1,536-well plates, reproducibility, incorporation into an automated screening setup and compatibility with currently available assay and detection methods are areas of concern (Janzen, 2014). In HCS, one of the biggest challenges to overcome will be the visualization of 3D structures with automated imaging systems. Optical light scattering, light absorption and poor light penetration with prolonged imaging acquisition times, and imaging of multicellular cultures and cells grown within complex geometrical structures, currently limit the applications of 3D cultures in HCS. One of the biggest challenges of incorporating 3D cultures into HTS will be to design systems that are compatible with liquid handling equipment. The hanging drop culture is the spheroid technology that has most advanced toward use in HTS. HDPs are available in 96- and 384-well formats but they require significant expertise in the use of the technology within a HTS setup. Collagen and Matrigel are commonly used hydrogels, but their natural origin limits the possibility of mimicking different tissue environments, the variations of different preparations impacts reproducibility, and their gelation properties prevent the handling at ambient temperature. Despite these challenges, HTS-compatible screening platforms are emerging. Synthetic matrices, while costly, have the advantages of providing defined, designed, and tunable material properties and allow for the controlled inclusion of biochemical cues. Self-assembling peptide hydrogels do not require covalent crosslinking reactions and can assemble into a defined hydrogel at physiological conditions. We have recently described an injectable hydrogel that flows under shear and is compatible with standard liquid automated handling equipment to form reproducible 3D cultures in 384-well plates (Worthington et al., 2017). The next step will be to build a 3D culture system that is versatile enough to enter mainstream drug discovery but can easily be fine-tuned to meet the tissue-specific characteristics of an *in vivo*-like microenvironment.

## Conclusions

The field of 3D cultures has grown exponentially in the past few years and offers considerable promise with broad applications in drug development and toxicity testing for a wide variety of diseases ranging from cancer to fibrosis to cardiac and neurological disorders. The major challenge will remain the creation of 3D cultures which are biologically relevant and recapitulate microenvironmental factors that resemble *in vivo* tissue and disease pathology. Given that the ECM alone has more than 300 biochemical constituents not including cellular components, this remains a daunting task. Nevertheless, with an increasing list of available 3D cell culture methods, we can take advantage of technologies that are most appropriate for a particular purpose such as mimicking a tumor environment or brain-specific matrix with appropriate tissue stiffness, recreation of a tissue barrier, or other specialized technical application. By combining biomedical engineering knowledge in the design of 3D scaffolds with knowledge of disease mechanism and biomarkers and genomic data, informed decisions can be made for the specific design of biomimetic scaffolds that most closely recapitulate factors promoting a particular disease phenotype, moving 3D drug discovery into the age of precision medicine.

## Author contributions

The author confirms being the sole contributor of this work and approved it for publication.

### Conflict of interest statement

The author declares that the research was conducted in the absence of any commercial or financial relationships that could be construed as a potential conflict of interest.

## Abbreviations

2D: Two-dimensional
3D: Three-dimensional
bFGF: Basic fibroblast growth factor
CNS: Central nervous system
ECM: Extracellular matrix
EGF: Epidermal growth factor
HA: Hyaluronan/hyaluronic acid
HCS: High-content screening
HDP: Hanging drop plate
HGF: Hepatocyte growth factor
HTS: High-throughput screening
IGF-1: Insulin-like growth factor 1
iPSC: Induced pluripotent stem cell
MMP: Matrix metalloproteinase
NGF: Nerve growth factor
PDGF: platelet-derived growth factor
PEG: Polyethylene glycol
TGF-β: Transforming growth factor-β
TIMP: Tissue inhibitor of metalloproteinase
VEGF: Vascular endothelial growth factor.
